# Effect of Epstein-Barr Virus DNA on the Incidence and Severity of Arthritis in a Rheumatoid Arthritis Mouse Model

**DOI:** 10.3389/fimmu.2021.672752

**Published:** 2021-05-10

**Authors:** Sukayna Fadlallah, Hadi Hussein, Mary-Ann Jallad, Marwa Shehab, Abdo R. Jurjus, Ghassan M. Matar, Elias A. Rahal

**Affiliations:** ^1^ Department of Experimental Pathology, Immunology, and Microbiology, American University of Beirut, Beirut, Lebanon; ^2^ Center for Infectious Diseases Research, American University of Beirut, Beirut, Lebanon; ^3^ Department of Anatomy, Cell Biology and Physiological Sciences, Faculty of Medicine, American University of Beirut, Beirut, Lebanon

**Keywords:** rheumatoid arthritis, C57BL/6J mice, chicken collagen type II, Epstein-Barr virus DNA, proinflammatory responses

## Abstract

**Objective:**

We recently demonstrated that EBV DNA is correlated with proinflammatory responses in mice and in rheumatoid arthritis (RA) patients; hence, we utilized an RA mouse model to examine whether EBV DNA enhances the risk and severity of arthritis and to assess its immunomodulatory effects.

**Methods:**

C57BL/6J mice were treated with collagen (arthritis-inducing agent), EBV DNA 6 days before collagen, EBV DNA 15 days after collagen, *Staphylococcus epidermidis* DNA 6 days before collagen, EBV DNA alone, or water. Mice were then monitored for clinical signs and affected joints/footpads were histologically analysed. The relative concentration of IgG anti- chicken collagen antibodies and serum cytokine levels of IL-17A and IFNϒ were determined by ELISA. The number of cells co-expressing IL-17A and IFNϒ in joint histological sections was determined by immunofluorescence.

**Results:**

The incidence of arthritis was significantly higher in mice that received EBV DNA prior to collagen compared to mice that only received collagen. Similarly, increased clinical scores, histological scores and paw thicknesses with a decreased gripping strength were observed in groups treated with EBV DNA and collagen. The relative concentration of IgG anti-chicken collagen antibodies was significantly increased in the group that received EBV DNA 6 days prior to collagen in comparison to the collagen receiving group. On the other hand, the highest number of cells co-expressing IFNϒ and IL-17A was observed in joints from mice that received both collagen and EBV DNA.

**Conclusion:**

EBV DNA increases the incidence and severity of arthritis in a RA mouse model. Targeting mediators triggered by viral DNA may hence be a potential therapeutic avenue.

## Introduction

Epstein-Barr virus (EBV) belongs to the *Herpesviridae* family and is considered one of the most prevalent viruses that affect humans (1, 2). It is estimated that more than 90% of the world’s population is seropositive for EBV (2). Upon infection, EBV establishes latency in B lymphocytes, which permits subsequent reactivation and recurrent infection. During these frequent recurrences EBV antigens including its DNA are shed (3). During childhood, EBV infection can be asymptomatic or may result in mild illness. However, if the infection is acquired during adolescence, it results in Infectious Mononucleosis (IM) (4). EBV has been associated with malignant lymphoproliferative diseases such as Burkitt’s lymphoma, epithelial carcinomas such as nasopharyngeal carcinoma, and Human Immunodeficiency virus (HIV)-related diseases such as hairy leukoplakia (5). Additionally, EBV has been linked with an increased risk of developing autoimmune diseases such as multiple sclerosis (MS), systemic lupus erythematosus (SLE), and rheumatoid arthritis (RA) (6).

Rheumatoid Arthritis (RA) is an inflammatory disorder that mainly affects joints and results in cartilage degradation and erosion, systemic complications, debility and early death (7). This disease is estimated to affect 1% of the population worldwide and occurs twice the rate in women compared to men (8). Although the cause of RA is unknown, a number of risk factors have been identified including environmental stimuli and genetic factors (9). Infectious agents are considered the undisputed leaders as environmental triggers (10–12). Particularly, EBV has been identified to be the most common potential environmental trigger for RA (13). Studies have showed elevated viral loads and increase in EBV-related autoreactive antibody levels in RA patients compared to healthy controls. EBV DNA/RNA have been identified in peripheral blood mononuclear cells (PBMC), synovial fluids, and synovial membranes in RA patients (14–17). Additionally, antibodies against Epstein–Barr nuclear antigen 1 (EBNA-1) and viral capsid antigen (VCA) have been detected at higher levels in sera and synovium of patients with RA compared to non-RA controls (18, 19). This results in decreased control of the virus, persistent exposure to EBV antigens and chronic inflammatory responses.

Although IL-17A plays an important role during bacterial and fungal infections, when produced in excess it is implicated in the pathogenesis of autoimmune diseases (20, 21). Several studies have demonstrated a pivotal role for IL-17A in inducing RA in humans. In patients with RA, high titers of IL-17A and their receptors have been detected in tissue extracts and synovial fluids (22). IL-17A plays a role in the differentiation of neutrophils, activation and cytokine release from neutrophils, fibroblasts, and monocytes, and triggering of Matrix Metalloproteases (MMP) (23, 24). Cells producing both IL-17A and IFNϒ have been implicated in autoimmunity and are detected at high levels in inflamed human and murine tissues (25). Hence, IL-17A has an essential role in the pathogenesis of RA by mediating pannus growth, matrix turnover, cartilage destruction, and osteoclastogenesis.

Like other members of the *Herpesviridae* family, EBV DNA is rich in unmethylated CpG motifs. Studies have shown that these CpG motifs present in the genomes of bacteria and some viruses, such as *Herpes simplex virus 1* (HSV-1) can activate the immune system promoting Th1-like responses (20, 26). Previous research by our group showed that EBV DNA induces proinflammatory responses, including IL-17A cytokine production, in mice, as well as triggers immune responses in flies (27, 28). Our group also demonstrated that increased EBV viral DNA loads in patients with rheumatoid arthritis (RA) correspond with higher levels of serum IL-17A. However, this correlation was not found in non-RA controls (29). Thus, with the study at hand we intended to examine the impact of EBV DNA on disease processes and evolution.This was done by assessing the effect of its DNA on the incidence and severity of arthritis in a mouse model of RA and determining the involvement of Th17 proinflammatory response.

## Materials and Methods

### Mice

The murine model for arthritis employed was the collagen-induced arthritis (CIA) in C57BL/6J mice (30–32). Female, 12-weeks of age mice, were obtained from the animal care facility at the American University of Beirut (AUB) and treated according to the Institutional Animal Care and Use Committee (IACUC) guidelines. They were co-housed in non-individually ventilated cages (non-IVC) in the same room. They had access to unlimited water and food. Mice were labeled with random ear tags and assigned to various groups; this was utilized for randomized assignment purposes.

### Determining the Effect of EBV DNA on the Incidence of Arthritis in the RA Mouse Model

**Figure 1:**
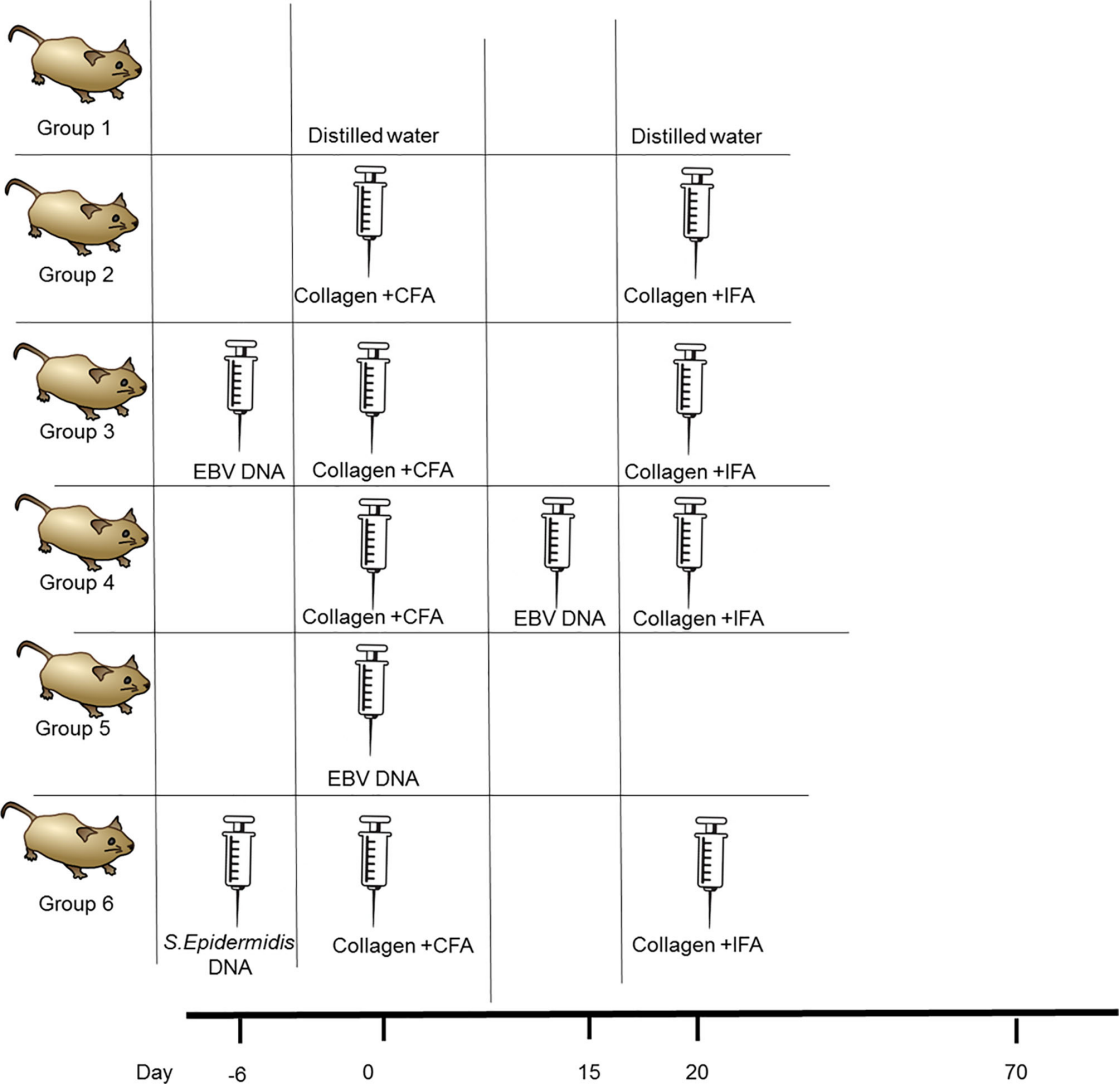
Treatment regimen for assessing the effect of EBV DNA in the type II chicken collagen-induced arthritis C57BL/6J mouse model. EBV, Epstein-Barr virus; CFA, complete Freund’s adjuvant; IFA, incomplete Freund’s adjuvant.

To assess if EBV DNA affects the incidence of arthritis in the CIA mouse model, female C57BL/6J mice were used. These mice were divided into five groups as shown in **Figure 1**. Induction of arthritis was performed as previously described (30, 33). The arthritis-inducing emulsion was produced by mixing an equal volume of type II chicken collagen (Chondrex, Inc, Redmond, Washington) with complete Freund’s adjuvant (CFA). To prepare CFA, heat-killed *Mycobacterium tuberculosis* (Invivogen, Toulouse, France), at a concentration of 3.3 mg/ml, was added to incomplete Freund’s adjuvant (IFA) (Chondrex, Inc, Redmond, Washington). Booster injections of type II chicken collagen were administered 20 days after the initial collagen challenge; these were given in a manner similar to that of the primary challenge but instead of CFA, IFA was used. The emulsion injections were given subcutaneously in the tail. The volume of both the eliciting agent primary and booster shots injected was 50 µl. C57BL/6J mice that received 50 μl of distilled water subcutaneously in the tail were included.

The study included an arthritis control group which received the inducing agent and the booster. One group was administered EBV DNA (Advanced Biotechnologies, Columbia, MD) 6 days prior to the primary challenge with collagen while another was administered the EBV DNA 15 days after the primary challenge with collagen. The days chosen for administration of EBV DNA were selected based on our previous observations that the pro-autoimmune cytokine IL-17A peaks in mouse sera 6 days after administration of the DNA (34). Hence, in the current study, the DNA was administered so peak levels of IL-17A would either coincide with collagen administration or with anticipated appearance of symptoms which occurs around 3 weeks after administration of the arthritis-inducing agent. Injections containing the viral DNA harbored 144×10^3^ copies of EBV DNA in 100 μl of distilled water and were given intraperitoneally. Mice that received 144× 10^3^ copies of EBV DNA in 100 μl of distilled water intraperitoneally were also examined. In addition, a control bacterial DNA-treated group was included which received 27.2 ng of *Staphylococcus epidermidis* DNA in 100 µl of water; this was administered 6 days prior to collagen (amount equivalent to 144 x 10^3^ copies of EBV DNA). Mice were then monitored for 70 days for the development of arthritis by assessing the ankle joint macroscopically for redness and swelling.

The relative concentration of IgG anti-chicken collagen antibodies was detected by ELISA. Commercially available microtiter plates were coated with Type II chicken collagen (Chondrex, Inc, Redmond, Washington). This was done by diluting the collagen stock (4 mg/ml in 0.05 M acetic acid) using 1X phosphate buffered saline (PBS) to a final concentration of 10 µg/ml. The chicken collagen was then added to the various wells in 100 µl aliquots, sealed with adhesive plastic cover, and incubated overnight at 4°C. The collagen coated plate was then washed 3 times with wash buffer (Abcam, Cambridge, UK). The plate was blocked with fetal bovine serum (FBS) for 30 minutes at room temperature. The sera of mice from the distilled water treated group, collagen-receiving group, and EBV DNA 6 days prior to collagen treated group were diluted 1:2 with FBS and transferred to their respective wells in 100 µl aliquots. The plate was then incubated for 2 hours at room temperature after which it was washed 3 times using the wash buffer. Horse radish peroxidase (HRP)-conjugated goat IgG anti-mouse antibody (Pierce, Waltham,USA) was diluted to 1/2000 with 0.1 M Tris buffered saline, pH 8, containing 25% FBS and 100 µl was added to each well. The plate was incubated for 2 hours at room temperature after which it was washed 3 times with the wash buffer. 3,3’, 5,5”-tetramethylbenzidine (TMB) was then added to each well (100 ul) and incubated ta room temperature in the dark for 10 minutes. A stop solution (Abcam) was then added to end the reaction. The absorbances of the various samples were then measured with an ELISA reader at a wavelength of 450 nm. The relative concentrations of the groups were normalized to the control group that received distilled water only.

### Assessing the Effect of EBV DNA on the Severity of Arthritis in the RA Mouse Model

At the end of the monitoring period, the hind paw thickness was measured using a caliper by placing it on either side of the ankle joint and measuring the width of the ankle joint from one side to the other. Additionally, the affected hind paws were clinically scored using a scoring system modified from that of (35) (**Table 1**). If a mouse had more than 2 paws that are affected, the sum of the scores were averaged to get the final score. The motor function of the affected joints was assessed using the grip strength meter (Ugo Basile, Gemonio (VA) Italy). The mouse was allowed to grip the metal bar with its affected paw while being pulled by its tail until it loses its grip. The peak gram force (gf) just before losing the grip is recorded and assessed in triplicates to obtain the average for each mouse.

**Table 1:** Clinical and histological scoring systems for arthritic C57BL/6J mice.

Paw clinical scoring							
**Paw Score**					**Clinical Observation**		
0						No redness and swelling	
0.25						Slight redness	
0.5						Slight redness and swelling	
0.75–1						Mild redness and swelling	
1.25-1.5						Moderate redness and swelling	
1.75–2						Severe redness and swelling	
**Ankle joint section histological scoring**							
**Ankle Joint Score**	**Cartilage Destruction**		**Edema**		**Inflammatory Infiltrate**		**Connective Tissue Disruption**
0	None		None		None		None
1	Mild		Mild		Mild		Mild
2	Moderate		Moderate		Moderate		Moderate
3	Severe		Severe		Severe		Severe
**Footpad section histological scoring**							
**Footpad Score**		**Edema**				**Inflammatory Infiltrate**	
0		None				None	
1		Mild				Mild	
2		Moderate				Moderate	
3		Severe				Severe	

Furthermore, histological assessment of affected joints/footpads was carried out. Joints/footpads were fixed in 10% formaldehyde, decalcified with Protocol™ Decalcifier B (Thermo Fisher Scientific, Waltham,USA), and embedded in paraffin. Sagittal sections were stained with hematoxylin and eosin. Ankle joints and footpads were scored based on the severity of a number of factors (**Table 1**). Blinded scoring was performed by two independent scorers.

### Assessing the Involvement of IL-17A and IFNϒ in the Response to EBV DNA in the RA Mouse Model

Following the sacrifice of mice 70 days post the initial collagen injection, blood was collected in EDTA blood collection tubes (BD, New Jersey, USA) from mice by cardiac puncture and centrifuged at 1500 rpm for 30 minutes to separate the serum. Subsequently, the levels of IL-17A and IFNϒ in the sera from arthritic mice from the various groups and non-arthritic mice from the control group was determined using a mouse IL-17A ELISA Kit and a mouse IFNϒ ELISA Kit (Abcam, Cambridge, UK).

The number of cells that were double positive for IL-17A and IFNϒ in joints from the various groups of mice was determined by immunofluorescence performed on histological sections of the ankle joints. Initially the sections were deparafinized by immersing the slides in xylol three times for a period of 5 minutes for each immersion. The sections were rehydrated in a series of decreasing concentrations of ethanol (100%, 95% and 75%) in two changes for 3 minutes each. Finally, the sections were placed in two changes of deionized water for 5 minutes each. Antigen retrieval was performed by placing the slides in citrate buffer (pH 6) for 90 minutes in a water bath at 60°C. The citrate buffer was prepared from 0.1M tri-sodium citrate dihydrate and 0.1M citric acid (18ml of citric acid and 82ml of tri-sodium citrate dihydrate brought to a final volume of 1L with deionized water) (36). The slides were then washed with Tris-buffered saline (TBS) (pH 7.4) after which the sections were permeabilized using 0.3% Triton X in 1x PBS. The samples were then washed three times in 1x PBS. After blocking the samples in 15% FBS in 1x PBS for 1 hr, the slides were incubated overnight with the fluorochrome-labeled antibodies antibodies Brilliant Violet 605 anti-mouse IL-17A (1:1500) and Pacific Blue 405 anti-mouse IFNϒ (1:1500) (Biolegend, California, USA) prepared in 1x PBS containing 15% FBS and 0.3% triton X. Sections were then washed twice with 1x PBS. The slides were then each covered with mounting solution and a coverslip then stored at 4°C. The mounting solution consisted of 80% glycerol, 223mM 1,4-diazabicyclo[2.2. 2]octane (DABCO), and 4mM Tris-HCl. Slides were observed using a Laser Scanning Confocal Microscope (Zeiss, Germany). The number of double positive cells per area was determined using ImageJ (NIH, Wisconsin, USA) and expressed as count per inch^2^.

### Statistical Analysis

Statistical analysis was performed using Graphpad Prism v6. The differences in the incidence of arthritis were analyzed using Fisher Exact Test. The comparisons in the histological and clinical scores were done using the Mann-Whitney U Test. Mean comparisons were analyzed using the two-tailed unpaired Student’s t test. P values less than 0.05 were considered statistically significant (37, 38).

## Results

### EBV DNA Increases the Incidence of Arthritis in the Chicken Collagen RA Mouse Model

The CIA murine model in C57BL/6J mice was used to assess the effect of EBV DNA on the development of arthritis. Several studies have indicated that the incidence of arthritis in this model ranges between 50% and 80% (33, 39, 40). Hence, the model allows observing the additive effect of EBV DNA on incidence.

**Figure 2:**
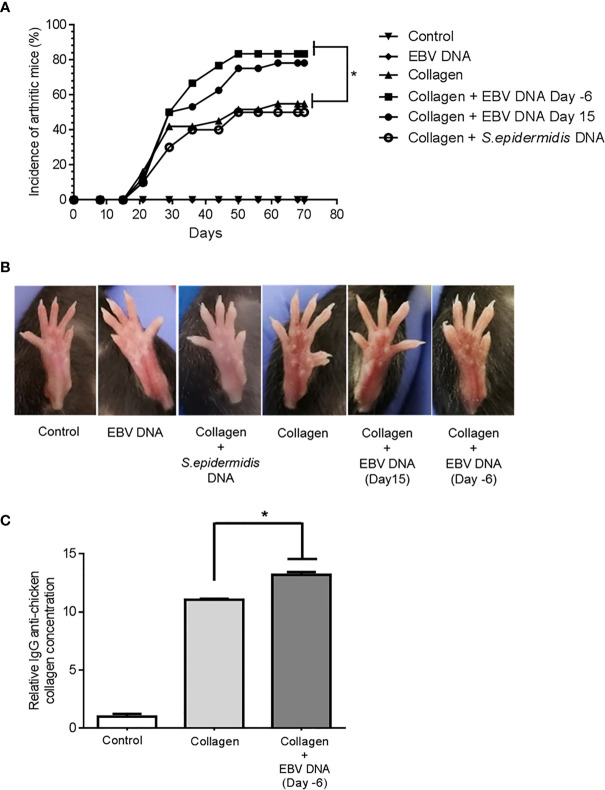
Effect of EBV DNA on the incidence of arthritis in the collagen-induced arthritis mouse model **(A)** Incidence (%) of arthritis in various groups of C57BL/6J mice treated with type II chicken collagen (n=31), EBV DNA 6 days before collagen (n=30), EBV DNA 15 days after collagen (n=32), *S. epidermidis* DNA 6 days prior to collagen (n=10), and control mice treated with distilled water (n=32) or EBV DNA only (n=8). **(B)** Representative images of hind paws from arthritis-affected animals. Redness and swelling in the paws and footpads are indicative of arthritis. **(C)** Relative concentration of IgG anti-chicken collagen antibodies in the arthritis groups (collagen only and EBV DNA 6 days prior to collagen mice) normalized to the distilled water control group. * indicates p< 0.05; comparisons were made to the group that received collagen only.

The incidence of arthritis was 54.8% in the collagen-receiving group (**Figure 2A**). Similarly, 50% of mice that received the bacterial control DNA in addition to collagen developed arthritis by the end of the monitoring period. On the other hand, the incidence increased in both groups that received EBV DNA in addition to collagen; however, this increase was only significant in the group that received EBV DNA 6 days prior to collagen (EBV DNA 6 days prior to collagen: 83.3%, p=0.0261; EBV DNA 15 days post collagen: 78.1%, p=0.0643). Symptoms of arthritis started appearing in the various groups at 3 weeks post-treatment with the inducing agent. Additionally, arthritic mice in the group that received EBV DNA 6 days prior to collagen showed symptoms within a narrower period (Days 21-50) of time in comparison to the group that only received collagen and the group that received EBV DNA 15 days post collagen (Day 21-62). **Figure 2B** shows representative ankle joints from arthritis-affected and control mice. The degree of redness and swelling in individual mice from the different groups was variable.

The role of anti-collagen antibodies as an initiating agent of RA has been studied extensively. In humans, 30-70% of RA patients have anti-type II collagen antibody responses depending on the stage of the disease (41–43). Additionally, several studies have shown that anti type II collagen-specific antibodies can play a role in triggering inflammation *in vivo* by several mechanisms (44, 45). In this study we measured the relative concentration of IgG anti-chicken collagen in the groups that had arthritis (collagen only and EBV DNA 6 days prior to collagen groups) and normalized these to that of the control group that received distilled water only. The fold change of the relative concentration of the anti-collagen antibodies was 11.06 and 13.31 fold in the collagen receiving group and EBV DNA 6 days prior to collagen treated group respectively compared to the distilled water control group (in both groups p= ≤ 0.0001). Additionally, the relative concentration of anti- collagen antibodies was significantly higher in the group that received EBV DNA 6 days prior to collagen compared to the group that received collagen only (p= 0.00054). **Figure 2C** shows the relative concentration of IgG anti-chicken collagen antibodies in the arthritis groups and the distilled water control group.

### EBV DNA Increases the Severity of Arthritis in the RA Mouse Model

Following the observation that EBV DNA increases the incidence of arthritis in the mouse model of RA, we determined the effect of EBV DNA on the severity of the disease. Thus, clinical assessments, motor function testing, and histological analyses were carried out.

**Figure 3:**
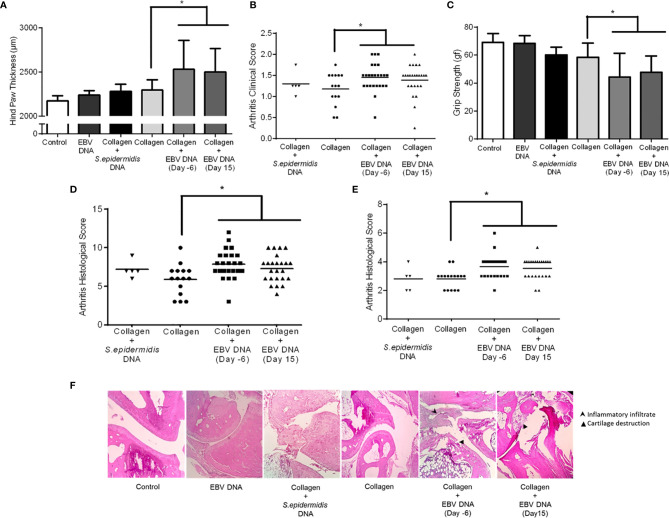
Effect of EBV DNA on the severity of arthritis in the collagen-induced arthritis mouse model. **(A)** Average hind paw thickness in C57BL/6J mice treated with distilled water, EBV DNA only, type II chicken collagen, *S. epidermidis* DNA 6 days prior to collagen, EBV DNA 6 days before collagen or EBV DNA 15 days after collagen. **(B)** Clinical scores of arthritic mice treated with *S. epidermidis* DNA 6 days prior to collagen, EBV DNA 6 days before collagen or EBV DNA 15 days after collagen, compared to mice treated with collagen only. **(C)** Average grip strength of arthritic animals compared to control mice treated with distilled water. **(D)** Histological scores of sections obtained from ankles of arthritic animals treated with *S. epidermidis* DNA 6 days prior to collagen, EBV DNA 6 days before collagen or EBV DNA 15 days after collagen compared to mice treated with collagen only. **(E)** Histological scores of sections obtained from footpads of arthritic animals treated with *S. epidermidis* DNA 6 days prior to collagen, EBV DNA 6 days before collagen or EBV DNA 15 days after collagen compared to mice treated with collagen only. **(F)** Representative histological sections of ankle joints from mouse groups. * indicates p<0.05.

The average affected hind paw thickness (**Figure 3A**) was 2172.22 µm in the distilled water group, 2237.5 µm in the EBV DNA receiving group, 2280 µm in the group receiving *S. epidermidis* DNA in addition to collagen, 2294.74 µm in the collagen-treated group, 2533.33 µm in the group receiving EBV DNA 6 days prior to the collagen (p=0.0048, compared to the collagen group) and 2500 µm in the group receiving EBV DNA 15 days after the collagen (p=0.0040, compared to the collagen group). The arthritis clinical scores in mice that received EBV DNA 6 days prior to collagen were significantly higher than in mice that only received collagen (p=0.0446) (**Figure 3B**). Moreover, 60% of the clinical scores of mice that received EBV DNA 6 days prior to collagen clustered at a score of 1.5 and above, whereas 60% to 80% of the scores of the mice that received collagen only or mice that received the bacterial control DNA in addition to collagen, respectively, were below 1.5.

Joint inflammation in mice induces grip strength deficit and loss of physical functionality (46). Thus, the grip strength in arthritic and healthy mice (basal levels in controls) was measured (**Figure 3C**). Results indicated that the average grip strength was 69.06 gf and 68.44 gf in the control groups (distilled water and EBV DNA group respectively). On the other hand, the grip strength decreased in all groups that had arthritis, however the largest drop was in the groups that received EBV DNA in addition to collagen (EBV DNA 6 days prior to collagen: 44.15, p=0.0496; EBV DNA 15 days post collagen: 47.79, p=0.0454, both compared to the collagen group).

Histological analysis of the affected ankle joints (**Figure 3D**) and footpads (**Figure 3E**) was carried out to observe tissue damage and inflammation. Concerning the ankle joints, around half of the mice from both groups that received EBV DNA in addition to collagen had a histological score of 8 or higher unlike mice that received collagen only (14.3%). In the footpad sections, 12.5% of mice had a score higher than 4 in the group that received collagen, whereas, 58.4% of mice had a score ≥4 in the groups that received EBV DNA in addition to collagen. Concerning mice that received *S. epidermidis* DNA in addition to collagen, only one mouse of the 5 that developed symptoms in this group had a score higher than 8 in the ankle joint scoring and a score higher than 4 in the footpad scoring. **Figure 3F** displays representative histological sections from ankles of affected and control mice. Hence, the histological sections from mice that received EBV DNA in addition to collagen at two time points show greater tissue damage and inflammation than mice that were injected with collagen.

### EBV DNA Increases Serum Proinflammatory Cytokines in an RA Mouse Model

After determining the effect of EBV DNA on the disease outcome in the RA mouse model used, we wanted to identify the proinflammatory cytokines that may be playing a role. As previously mentioned, IL-17A and IFNϒ play a major role in the development of arthritis. Thus, levels of these cytokines were determined in the sera of arthritic mice from the various groups in addition to those in the vehicle-treated control group. Since the increase in the incidence of arthritis in the group of mice that received EBV DNA 15 days post-collagen was not significant, this group was not included in our cytokine assessments. Additionally, since the group that received *S. epidermidis* DNA in addition to collagen did not show a similar incidence and severity of arthritis in comparison to the collagen-receiving group, the immune response in these mice was not assessed.

**Figure 4:**
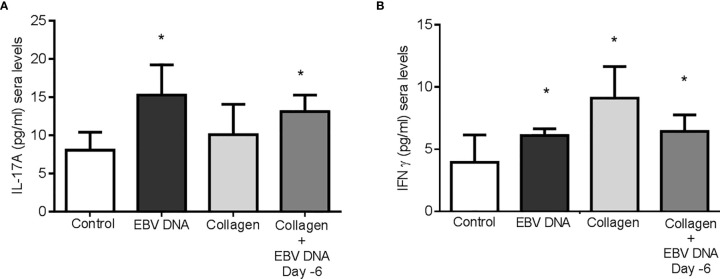
Effect of EBV DNA on proinflammatory immune response in the collagen-induced arthritis mouse model. **(A)** Serum IL-17A levels in arthritic animals from C57BL/6J mouse groups treated with distilled water, EBV DNA only, type II chicken collagen or EBV DNA 6 days before collagen compared to control mice treated with distilled water. **(B)** Serum IFNϒ levels in arthritic animals compared to control mice treated with distilled water. * indicates p<0.05.

Treatment of mice with collagen only or EBV DNA 6 days prior to collagen led to a significant increase in IFNϒ sera levels (p=0.003 and p=0.0399 respectively) when compared to mice injected with distilled water. However, mice that were treated with either EBV DNA only or EBV DNA 6 days prior to collagen showed a significant increase in IL-17A (p=0.003 for both) (**Figures 4A, B**). These results indicate that arthritic mice have higher levels of these proinflammatory cytokines. IL-17A was higher in the group that received EBV in addition to collagen than the group that was given collagen only but this increase was not significant (p= 0.134), whereas IFNϒ was higher in the latter (p= 0.045). This indicated that differences in systemic levels of these cytokines were not sufficient to explain the increased incidence and severity in the group receiving both collagen and EBV DNA.

**Figure 5:**
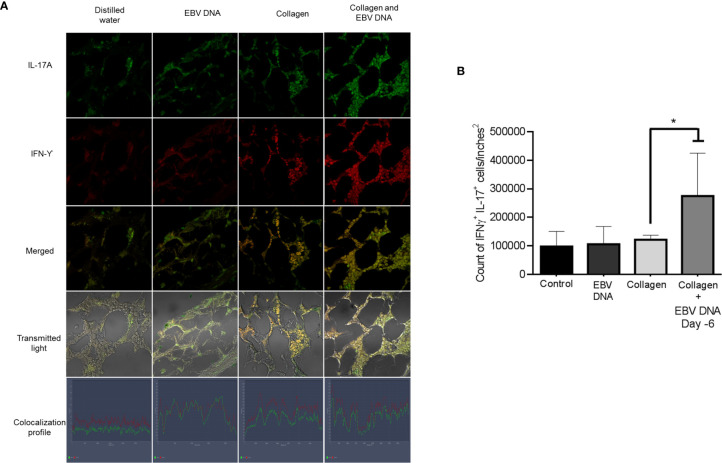
Effect of EBV DNA on IFNY^+^IL-17^+^ cells in the collagen-induced arthritis mouse model. **(A)** Immunofluorescent staining for IL-17A and IFNY in ankle joint sections from arthritic animals in C57BL/6J mouse groups treated with EBV DNA, type II chicken collagen, EBV DNA 6 days before collagen or distilled water. **(B)** IFNY^+^ IL-17^+^ cell counts in joints of arthritic animals; * indicates p < 0.05 compared to mice treated with collagen alone.

Hence, we identified the number of cells positive for both IL-17A and IFNγ in the affected joint ankles and their controls. Immunofluorescence and confocal microscopy imaging were carried out for this purpose (**Figure 5A**). The highest count of IL-17A and IFNϒ double positive cells was identified from histological sections obtained from mice that received EBV DNA 6 days prior to collagen in comparison to all groups (p=0.022 *vs* control, 0.032 *vs* collagen, 0.029 *vs* EBV DNA only). Joint sections from mice that received collagen only or EBV DNA only had a higher number of cells that were positive for both L-17A and IFNϒ in comparison to the control group; however, this increase was not significant (p=0.322 collagen vs control, 0.821 EBV DNA *vs* control) (**Figure 5B**). These observations were further supported by the colocalization profile. It showed that the highest intensity of both markers was in the group that received EBV DNA in addition to collagen. The level of colocalization in the group of mice that received EBV DNA only was lower than in both groups of mice that had arthritis. A low level of intensity was determined in the group that received distilled water.

## Discussion

Viruses and bacteria are considered the main environmental challenges that trigger an inappropriate immune response that results in an autoimmune disease such as RA. EBV DNA, shed during the common EBV-reactivated infection, may influence the development of autoimmune diseases by increasing the production of proinflammatory cytokines. Previous studies by our group indicated that EBV DNA enhances the production of proinflammatory cytokines in wild type mice (34). In the study at hand we employed a mouse model of arthritis to demonstrate that these proinflammatory properties of EBV DNA directly contribute to the development and severity of an autoimmune disease, in particular RA.

CIA is a conventional model of RA that is generated by active immunization (47, 48). This mouse model has many characteristics similar to human RA including breach of tolerance and generation of autoantibodies toward self and to collagen, which makes it the golden standard for *in vivo* models of RA (49). Collagen type II, the only form of collagen that induces acute or subacute polyarthritis in this model, is the main type in articular cartilage. In some RA patients, immunoreactivity to collagen type II has been identified (50, 51). DBA/1 mice are the most commonly used strains in CIA since it is the most susceptible strain (52). In these mice, arthritis can be induced in 80–100% of the animals. Since our aim was to determine the additive effect of EBV DNA in the development of RA, it was necessary to choose a mouse strain with a moderate arthritis response to an inducing agent. Hence, CIA in C57BL/6J mice was used for this purpose. Several studies have indicated that the incidence of arthritis in this model ranges between 50% and 80%, providing a margin to observe the EBV DNA effect (33, 39, 40). The model in C57BL/6J mice also results in a milder, however more chronic disease, with prolonged T cell responses (30). Additionally, these mice usually develop arthritis 3-8 weeks post immunization. Moreover, type II collagen from chicken is needed specifically to induce arthritis in this model, as C57BL/6J mice are resistant to type II bovine collagen (53, 54).

Similar to other studies (40), the incidence of arthritis in the group of mice that received collagen only in our study was 54.8%. When EBV DNA was administered in addition to collagen, the incidence of the disease increased to 83.3%. This was in accordance with our previous studies demonstrating that EBV DNA increases proinflammatory cytokine levels in mice leading to systemic inflammation and acting as a risk factor for the development of autoimmune diseases. Additionally, the group that received the bacterial control DNA in addition to collagen had a similar incidence to the group that received collagen only. This indicates that the increase in incidence seen in the group that received EBV DNA in addition to collagen was due to the immuno-stimulatory effect of the EBV DNA. The group that received EBV DNA 6 days prior to collagen had a higher incidence of arthritis than the group that received EBV DNA 15 days post collagen. Additionally, the increase in incidence of the disease in the latter group was not significant when compared to the control groups. We previously observed that IL-17A levels peak 6 days after DNA administration. The significantly higher incidence observed in the group that received EBV DNA 6 days prior to collagen is hence likely the result of an EBV DNA-triggered IL-17A response early in the arthritis-induction protocol.

It has been well documented that the generation of collagen type II-specific antibodies is required for RA development and progression. A study by Svensson et al. (55) showed that B cell deficient mice with a susceptible background to CIA do not develop arthritis when induced with collagen. Additionally, administration of anti-collagen antibodies induce arthritis in DBA1 mice (56). In our study it was shown that mice that developed arthritis had a significantly higher relative concentration of IgG antibodies against type II chicken collagen than mice treated with distilled water only. Additionally, it was demonstrated that mice that received EBV DNA 6 days prior to collagen had an increased relative concentration of these antibodies in comparison to mice treated with collagen only. In a series of *in vivo* studies it was shown that the antibody mediated immune response against collagen is one of the major mechanism of arthritis induction. This might occur when anti-type II collagen antibodies bind to normal joint cartilage surface. An inflammation cascade is then triggered by these antibodies by forming immune complexes stimulating the complement system or through direct engagement of cells carrying Fc receptors (44, 45, 57, 58).

Previous studies have demonstrated a pathologic role for IL-17A in mouse models of arthritis by neutralizing this cytokine (59) or using IL-17A deficient mice (60). Additionally, IL-17A has been shown to play a primary role in CIA by priming collagen specific T and B cells (60). In our study, IL-17A sera levels were increased in all arthritic mice (collagen and collagen with EBV DNA) in comparison to the distilled water-treated group. The highest increase in IL-17A levels were observed in the groups that received EBV DNA only and EBV DNA in addition to collagen. Lubberts et al. (59) have indicated that IL-17A not only plays a role in the initial phase of the disease by inducing inflammation and tissue destruction, but also functions in the later phases by prolonging and propagating the disease.

The role of IFNϒ in CIA is contradictory with different studies suggesting both pathogenic and protective effects (61). Its pathogenic roles include: 1) potent activation of macrophages and neutrophils, 2) upregulation of the expression of MCH-II on APCs including cells that do not express this molecule thus playing a role in development of autoimmune diseases 3) induction of differentiation of Th0 cells to Th1 which in turn produce IFNϒ hence creating a positive feedback loop and 4) stimulation of leukocyte trafficking and chemokine production. On the other hand, its protective roles include: 1) inhibition of Th17 differentiation and of IL-17A effector functions 2) increased activity of Treg cells 3) Inhibition of processes enabling macrophages and monocytes to give rise to osteoclasts and 4) reduction of neutrophil mobilization (61). In our study, the IFNϒ sera levels were increased in arthritic groups. However, the highest increase in this cytokine was in the group that received collagen only. In line with our results, the study by Inglis et al. (30) showed that IFNϒ levels in lymph node cells in the C57BL/6J CIA model were high both in the early and late phase of the disease. The level of IFNϒ was lower in the group that received EBV DNA in addition to collagen than the group that received only collagen. This might indicate that in the former group the cytokine profile balance may be shifted from a predominantly Th1 to a Th17 immune response.

As indicated above, the role of IFNϒ and IL-17A in autoimmune and inflammatory diseases is well-documented. However, little information is known about the interplay of these cytokines in autoimmune diseases. Several studies have shown that IL-17A^+^IFNϒ^+^ T cells were elevated in various autoimmune diseases. A study by Kebir et al. (62) demonstrated that there was an increased tendency for lymphocytes obtained from the blood of multiple sclerosis patients to expand into IFN‐γ–producing Th17 cells. Additionally, a large number of lymphocytes that are double positive for these cytokines were identified in the brains of these patients. Annunziato et al. (63) showed the presence of a subset of Th17 cells that produce both IFNϒ and IL-17A in the gut of patients with Crohn’s disease. Our study shows that the highest counts of IL-17A^+^ IFNϒ^+^ cells are seen in joints from mice given EBV DNA in addition to collagen. These mice also displayed the severest symptoms; this supports previous findings indicating that cells that produce both IFNϒ and IL-17A are more cytotoxic and potent (25). The systemic levels of IFNϒ and IL-17A were elevated in both the group that received collagen only and the group that received EBV DNA and collagen; however, there were no marked differences in the systemic levels of the cytokines between the two groups. Hence, the increase in the incidence and severity of arthritis in the group that received both the viral DNA and collagen is not explained by the systemic levels of these cytokines. On the other hand, both the anti-collagen antibodies and the joint IL-17A^+^ IFNϒ^+^ cells were significantly elevated in the group that received both EBV DNA and collagen. Therefore, the enhanced levels of the arthritis-triggering antibodies coupled with the increase in localized inflammation in the joints is what likely led to this increase in incidence of arthritis and exacerbation of its symptoms.

In conclusion, our study indicates that EBV DNA enhances the incidence of arthritis in mice and exacerbates the disease. EBV DNA also increases the number of IL-17A^+^IFNϒ^+^ T cells in joints of arthritic mice. A recent study by our group showed that the endosomal Toll-like receptors (TLRs) 3, 7 and 9 are involved in the EBV DNA-mediated triggering of IL-17A production in mice (64). Hence, targeting these receptors may be of therapeutic or prophylactic value in subjects with RA or at risk of a flare-up. Given the ubiquity of EBV infection, a large proportion of RA-affected individuals may benefit from such approaches. A better understanding of the various factors involved in the development of RA will possibly help in creating individualized treatments, which might include targeting mediators triggered by viral DNA.

## Data Availability Statement

The original contributions presented in the study are included in the article/supplementary material. Further inquiries can be directed to the corresponding author.

## Ethics Statement

The animal study was reviewed and approved by IACUC - American University of Beirut.

## Author Contributions

SF: Data curation, Formal analysis, Methodology, Investigation, Writing – original draft. HH: Data curation, Investigation. M-AJ: Data curation, Investigation. MS: Data curation, Investigation. AJ: Methodology, Investigation. GM: Investigation, Writing – review & editing. ER: Formal analysis, Funding acquisition, Methodology, Investigation, Project administration, Resources, Supervision, Validation, Writing – review & editing. All authors contributed to the article and approved the submitted version.

## Funding

This research was funded by the Asmar Fund. The funder played no role in the study design, collection, analysis, and interpretation of the results or writing the manuscript.

## Conflict of Interest

The authors declare that the research was conducted in the absence of any commercial or financial relationships that could be construed as a potential conflict of interest.
